# MiR-27a Regulates Apoptosis in Nucleus Pulposus Cells by Targeting PI3K

**DOI:** 10.1371/journal.pone.0075251

**Published:** 2013-09-25

**Authors:** Gang Liu, Peng Cao, Huajiang Chen, Wen Yuan, Jianxi Wang, Xianye Tang

**Affiliations:** Department of Orthopaedic Surgery, Changzheng Hospital, Second Military Medical University, Shanghai, P. R. China; School of Medicine and Health Sciences, University of North Dakota, United States of America

## Abstract

The precise role of apoptosis in the pathogenesis of intervertebral disc degeneration (IDD) remains to be elucidated. We analyzed degenerative nucleus pulposus (NP) cells and found that the expression of miR-27a was increased. The overexpression of miR-27a was further verified using real-time RT-PCR. Bioinformatics target prediction identified phosphoinositide-3 kinases (PI3K) as putative targets of miR-27a. Furthermore, miR-27a inhibited PI3K expression by directly targeting their 3’-UTRs, and this inhibition was abolished by mutation of the miR-27a binding sites. Various cellular processes including cell growth, proliferation, migration and adhesion are regulated by activation of the PI3K/AKT signaling pathway, and nucleus pulposus cells are known to strongly express the phosphorylated survival protein AKT. Our results identify PI3K as a novel target of miR-27a. Upregulation of miR-27a thus targets PI3K, initiating apoptosis of nucleus pulposus cells. This present study revealed that downregulated miR-27a might develop a novel intervention for IDD treatment through the prevention of apoptosis in Nucleus pulposus Cells.

## Introduction

Intervertebral disc degeneration (IDD) is considered to be the pathological basis of spinal degenerative diseases, which bring a global burden with severe healthcare and socioeconomic costs [1,2]. The mechanisms underlying IDD remain largely unknown, although a variety of factors have been suggested to influence its etiology. These include age, genetics, systemic factors and toxins, all of which can affect the balance of biochemical reactions leading to disc degeneration [3,4]. Mechanical factors such as compressive loading, shear stress and vibration have also been implicated [5,6]. However, the cause of the increase in apoptosis remains a mystery.

The intervertebral disc (IVD) is composed of an outer fibrous layer, the annulus fibrosus, and an inner gelatinous core rich in proteoglycans, the nucleus pulposus (NP). IVD cells, especially NP cells, are important in maintaining the integrity of the IVD through their role in producing type II collagen, aggrecan and other components of the extracellularmatrix (ECM). It has been reported that the central features of IDD are the reduction in the NP cell population and the loss of ECM [7]. A more recent study showed that NP cells possess distinct characteristics which are necessary for IVD homeostasis [8], while a variety of other studies both *in vitro* and *in vivo* suggest that an important contributor to the development of IDD is the cellular loss due to excessive apoptosis of disc cells [9,10].

One clue to the mechanism is that nucleus pulposus cells exhibit a robust expression of the phosphorylated survival protein AKT [11]. Activation of phosphoinositide-3 kinase (PI3K)/AKT signaling regulates cell growth, proliferation, migration and adhesion [12,13]. This death receptor pathway represents the extrinsic apoptotic signaling pathway, whereas the intrinsic pathway originates from the mitochondria and involves the activation of Bcl-2 family members [14]. Both the extrinsic and intrinsic pathways are known to play essential roles in the apoptosis of human NP cells [15].

There is a growing body of evidence in support of the hypothesis that the newly deﬁned small noncoding RNAs, miRNAs, regulate the apoptotic machinery. The control of gene expression by miRNAs involves targeting of mRNAs leading to either repression of translation or RNA degradation. To date, miRNAs have been identified as key regulators in proliferation, differentiation, apoptosis, organ development, inﬂammatory diseases and a wide range of other biological processes [16-19].

As a multi-functional miRNA, miR-27a is expressed in diverse tissues. The aberrant expression of miR-27a has been found to be associated with a variety of diseases [20-22]. Moreover, Zhao et al. showed that miR-27a is important in cell proliferation, apoptosis, and tumorigenesis [23]. Yang et al. found that miR-27a affects the apoptotic signaling pathway in glioma initiation and progression [24]. Given that miR-27a is crucially involved in apoptotic pathways, as well as disorders characterized by abnormal apoptosis, we hypothesized that miR-27a might play a role in the process of IDD. To date, few studies have addressed the etiology of IDD from the perspective of miRNAs. Accordingly, the aim of this study was to investigate the role of miR-27a in IDD and to further clarify the underlying mechanisms of IDD.

## Methods and Materials

### Isolation and Primary Culture of Human NP cells

The Institutional Review Broad of second Military Medical University, Shanghai, China, approved this study with written informed consent obtained from each patient. Thirty-two Human NP specimens were collected from patients with idiopathic scoliosis [n=20; average age 21.4 (range 18–38) years] and not disc herniation, and therefore contact between these tissues and cells outside of the disc, these are macrophages, endothelial cells and other immune cells, were minimal or nonexistent. No granulation tissue was present. Two cell types populate the human NP: notochordal cells and chondrocyte-like cells. As the notochordal cells slowly decline in abundance and appear to be absent after 10 years of age, NP tissues of human adolescents and adults consist of only chondrocyte-like cells. Tissues specimens were first washed twice with PBS, NP was separated from the AF using a stereotaxic microscope, then cut into pieces (2–3 mm^3^), and NP cells were released from the NP tissues by incubation with 0.25 mg/ml type II collagenase (Invitrogen) for 12 h at 37°C in Dulbecco’s modified Eagle medium (DMEM; GIBCO, Grand Island, NY). After isolation, NP cells were resuspended in DMEM containing 10% FBS (GIBCO, NY, USA), 100 µg/ml streptomycin, 100U/ml penicillin and 1% L-glutamine, and then incubated at 37°C in a humidified atmosphere with 95% air and 5% CO_2_. The confluent cells were detached by trypsinization, seeded into 35-mm tissue culture dishes in complete culture medium (DMEM supplemented with 10% FBS, 100 µg/ml streptomycin and 100U/ml penicillin) in a 37°C, 5% CO_2_ environment. The medium was charged every 2 days. NP cells cultured in vitro within 10 days, the second passage was used for subsequent experiments.

### Establishment of a model of compression induced apoptosis in nucleus pulposus cells

Primary cultures of NP cells were divided into two groups, the experimental group was given artificial compressed air (0.5% CO_2_ + 99.5% compressed air) in a pressurized cabin, and the control group was cultured in a 1.0 MPa incubator.

### Apoptosis assay: ﬂow cytometry (FCM)

The cells were lifted using a 0.25% trypsin/EDTA (Invitrogen) from the tissue culture plates for Flow cytometric analyses. Apoptotic NP cells were identified by staining with FITC Annexin V/PI (BD Biosciences, San Diego, CA)，and then analyzed by FCM. In brief, after washing twice with PBS, 1×10^6^ cells were resuspended in binding buffer (10 mM HEPES, pH 7.4; 140 mM NaCl; 2.5 mM CaCl2). PI (400 µL of 50 µg/mL) was added and the cells were incubated at room temperature for 10 min before analysis.

### MTT assay

NP cells were plated in 96-well plates at 2,000 cells per well in 120 µL of cell culture medium and incubated at 1.0 MPa for various times (0, 12, 24, 36 h). After incubation for the appropriate time, the cells were incubated with 20µL MTT (at a ﬁnal concentration of 0.5 mg/mL) at 37°C for a further 4 h. The medium was then removed and the precipitated formazan was dissolved in 150 µL DMSO. After shaking for 10 min, the absorbance at 570 nm was detected using an lQuant Universal Microplate Spectrophotometer (Biotek Instruments). Each experiment was repeated at least three times.

### Immunohistochemistry

Tissues were fixed in 10% neutralized formalin and embedded in paraffin blocks. Sections (4µm) were then prepared for immunohistochemical examination. After deparaffinizationand rehydration, antigen retrieval was performed by boiling in 10 mmol/Lof citrate buffer (pH 6.0) for 10 min. Endogenous peroxidase activity was blocked by soaking for 30 min inmethanol containing 0.3% H_2_O_2_, then the sections were blocked with 2% bovine serum albumin in PBS for 30 min before incubating with mouse anti-human PTEN monoclonal antibody (Abcam, dilution 1:500). The immune complex was visualized usingthe DakoREAL™EnVision™ Detection System, Peroxidase/DAB, Rabbit/Mouse (Dako), according to the manufacturer’s protocol. The cytoplasm was counterstained with hematoxylin.

### RNA isolation

Total RNA from cryostat tissues or isolated cells was isolated usingTRIzol (Invitrogen) and a mirVana™PARIS ^™^Kit (Applied Biosystems, Foster City, CA) according to the manufacturer’s instructions. RNA integrity was monitored by electrophoresis in 8% denaturing polyacrylamide gels.

### Real-time PCR

RT-PCR for miR-27a in NP cells was performed using TaqMan miRNA assays. In brief, miRNAs were reverse-transcribed to cDNAs, using a high-capacity cDNA Archive Kit (ABI, Foster City, CA). MiR-27a speciﬁc stem–loop RT primers were used according to the manufacturer’s instructions (Human miRNAmicroarrays, Agilent Technologies). RNA concentrations were determined using a NanoDrop instrument (NanoDrop, Wilmington, DE). The levels of miR-27a were normalized to U6 controls. All RT reactions, including U6 controls, were run in triplicate in a GeneAmp PCR 9700 Thermocycler (ABI). The relative amounts of miR-27a were calculated using the RelativeExpreSSion,RQ

### PIK3CD 3’-UTR vector construction and luciferase reporter assay

The 3’ UTR of human PIK3CD containing the miR-27a binding site was PCR-amplified from NP cells genomic DNA (PCR primers, sense: 5’- TCC GTG AGA GCT GGA AAA CC -3’ and antisense: 5’- TGG TTC TAA CTA ACT CCA GCC G -3’, Product length 2060bp) and inserted into the XbaI and FseI sites of pGL3-control (Promega, Madison, WI), downstream of the stop codon of the firefly luciferase reporter gene, respectively. HEK 293 cells were co-transfected with 0.8 µg firefly luciferase reporter vector containing the target site, 100 nM miR-27a double-stranded mimics or miR-control (Ambion, Austin, TX) and 0.04 µg renilla luciferase control vector (pRL-CMW-Promega, Madison, WI), using Lipofectamine 2000 (Invitrogen). Assays were performed 36h after transfection, using the dual luciferase reporter assay system (Promega). Firefly luciferase activity was normalized to renilla luciferase activity. The mutation on miR-27a binding sites in human PIK3CD 3’-UTR were generated using the Quick Change XL Site-Directed Mutagenesis kit (Stratagene, La Jolla, CA). Mutations consisted of replacing three consecutive base pairs at the 3’ region of the site.

### Up-regulation of miR-27a

The lentiviral vector labeled with green fluorescent protein (GFP) encoding Has-miR-27a, together with a scrambled sequence as the control, was purchased from Invitrogen. Human NP cells were plated at a density of 1.5×10^5^ cells/well in a 24-well plate in a final volume of 250µL complete medium. Viral solutions at a multiplicity of infection (MOI) of 10 were added to NP cells. Following 5 h of incubation at 37°C, the cells were allowed to recover over the ensuing 96 h in culture medium. To verify cell transfection, culture flasks were inspected by fluorescence microscopy. Subsequently, cells were evaluated by western blot, using standard protocols. In brief, proteins were extracted using RIPA lysis buffer and electrophoresed on NuPage gels (Invitrogen), using 100 µg protein/sample. The proteins were transferred to a polyvinylidene difluoride membrane (Invitrogen) and blocked in PBS containing 5% fat-free milk powder. Antibodies against PIK3CD were used to detect the blotted proteins. Goat anti-rabbit immunoglobulin conjugated to horseradish peroxidase (Sigma, St. Louis, MO) was used as the secondary antibody. The immunoreactive proteins were detected using the Pierce ECL western blotting substrate (Pierce Biotechnology, Rockford, IL).

### Knockdown of miR-27a

The lentiviral vector labeled with GFP encoding antigomiR-27a and lentiviral vector control was purchased from Invitrogen. The knockdown of miR-27a in cultured NP cells was achieved by transfection with lentiviral antigomiR-27a with a MOI of 10, as described above. PIK3CD activities were analyzed by western blot as described. To knock down the PIK3CD, control siRNA (cat. sc-37007 Santa Cruz Biotechnology, Inc., Santa Cruz, CA, USA) or human PIK3CD siRNA (cat. sc-39131, Santa Cruz, Biotechnology, Inc.) were transfected into NP cells using Lipofectamine 2000 (Invitrogen).

### Statistical analysis

Comparisons of two-group parameters were performed using Student’s t-test. Comparisons of multiple group data were performed using one-way analysis of variance followed by Tukey’s post-hoc test. Differences with *P*<0.05 were considered statistically significant. Statistical analysis was performed using the SPSS statistical package (SPSS, Chicago, IL).

## Results

### Compression induces apoptosis of NP cells

**Figure 1:**
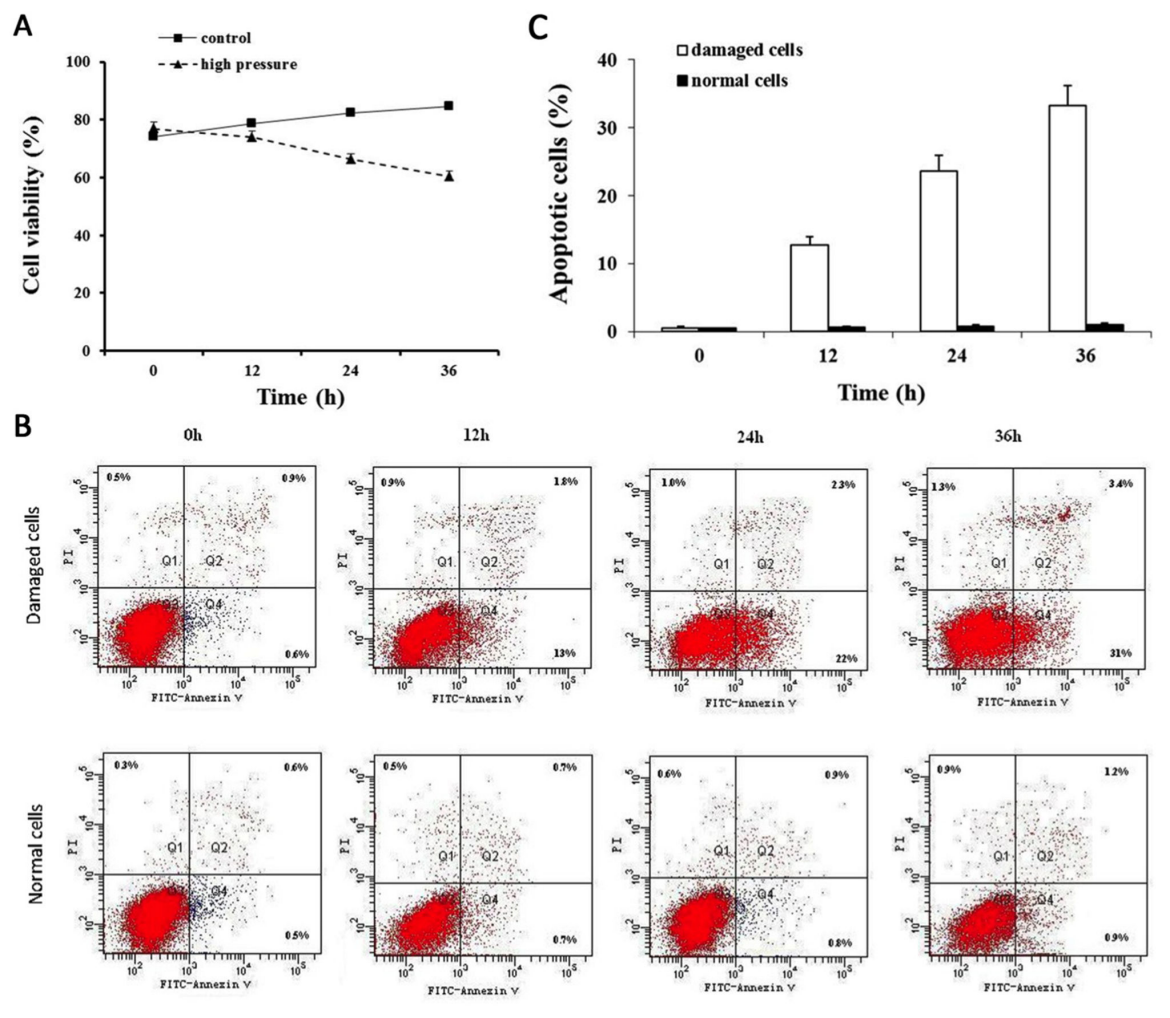
Apoptosis was increased in the injury model. (A) Assessment of cell viability by MTT. The cell viability was compared in the different NP cell groups (∗P <0.05). Data are representative of three experiments; error bars represent SEM. (B) Contour diagram of FITC-Annexin V/PI FCM of human NP cells. The graphs represent typical results of cell apoptosis; values represent the means of two experiments. (C) Results of cellular apoptosis was expressed as a fold change. Results are shown as mean ± SEM. Data are representative of three independent experiments (*P<0.05, **P<0.01).

To examine the regulatory effect of compression on the growth of NP cells, the MTT assay was used as a measure of cell viability. As shown in Figure 1A, compression inhibited the viability of NP cells in the pressure groups in a notably time-dependent manner. To further understand whether the reduced cell viability was due to apoptosis, we studied apoptosis using the Annexin V/PI assay. We observed a time-related increase in cell apoptosis in all experimental groups (Figure 1B, C). Similarly, the percentage of cells with apoptotic nuclei, indicated by PI staining, significantly increased in the experimental groups compared to the controls.

### miR-27a is up-regulated in damaged NP cells

**Figure 2:**
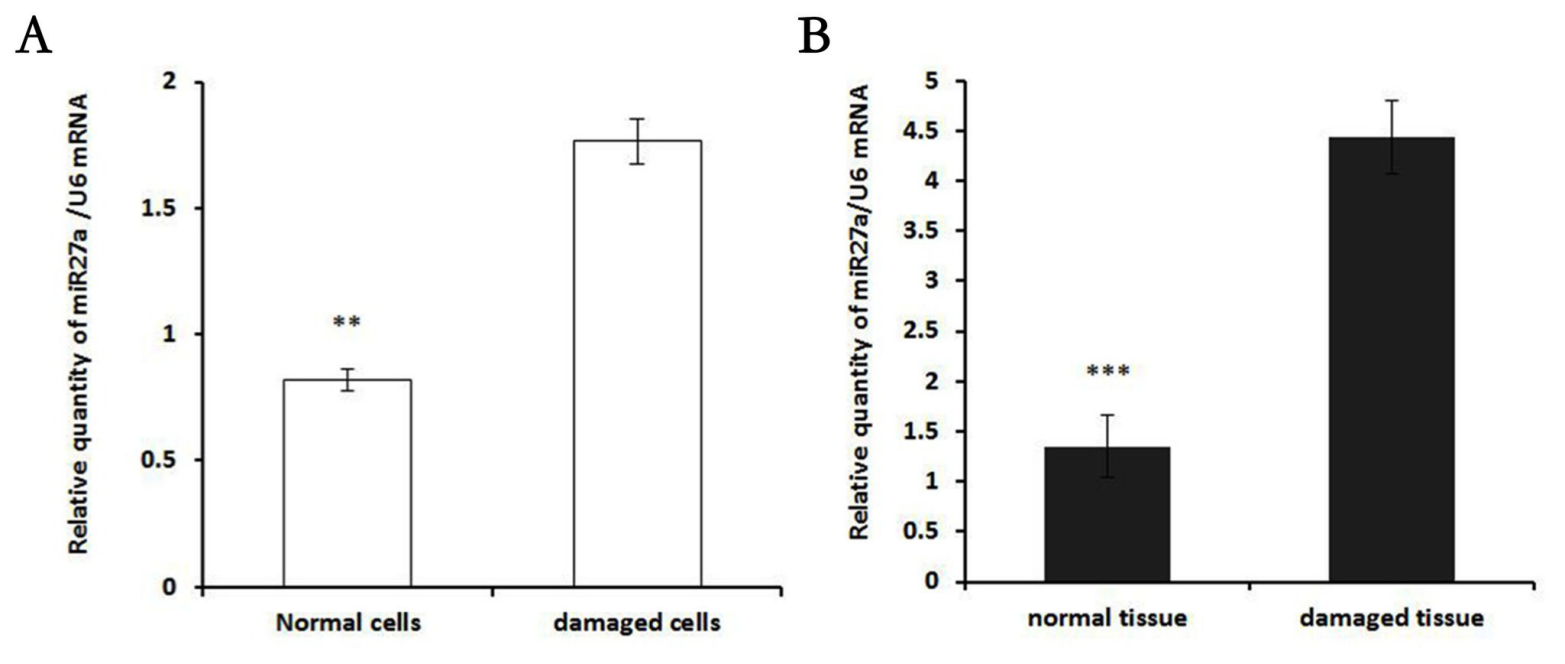
The expression levels of miR-27a were analyzed in human degenerative NP compared with control NP by real-time RT-PCR. The relative expression of miR-27a was normalized to the endogenous control U6. Each sample was analyzed in triplicate (**P <0.01, ***P<0.001). (A) miR-27a is up-regulated in damaged NP *in* *vitro*. (B) miR-27a is up-regulated in damaged NP cells *in* *vivo*. Data are representative of six independent experiments. Error bars represent SEM; *P<0.05.

As a multi-functional miRNA, miR-27a is expressed in diverse tissues, and aberrant expression of miR-27a is found to be associated with various diseases. The expression levels of miR-27a were analyzed in human degenerative NP compared with control NP by real-time RT-PCR. As shown in Figure 2, in vitro, miR-27a was significantly up-regulated (*P*<0.05) in damaged cells, while miR-27a was significantly up-regulated (*P*<0.05) in NP cells from individuals with of IDD in vivo, an observation which was confirmed by semi-quantitative RT–PCR (Figure 2A, B). Given the unique hallmarks of miR-27a and its roles in regulating apoptotic pathways, we selected miR-27a for further investigation to shed more light on the etiology of IDD.

### miR-27a inhibits PIK3CD expression and regulates apoptosis by directly targeting their 3’-UTRs

**Figure 3:**
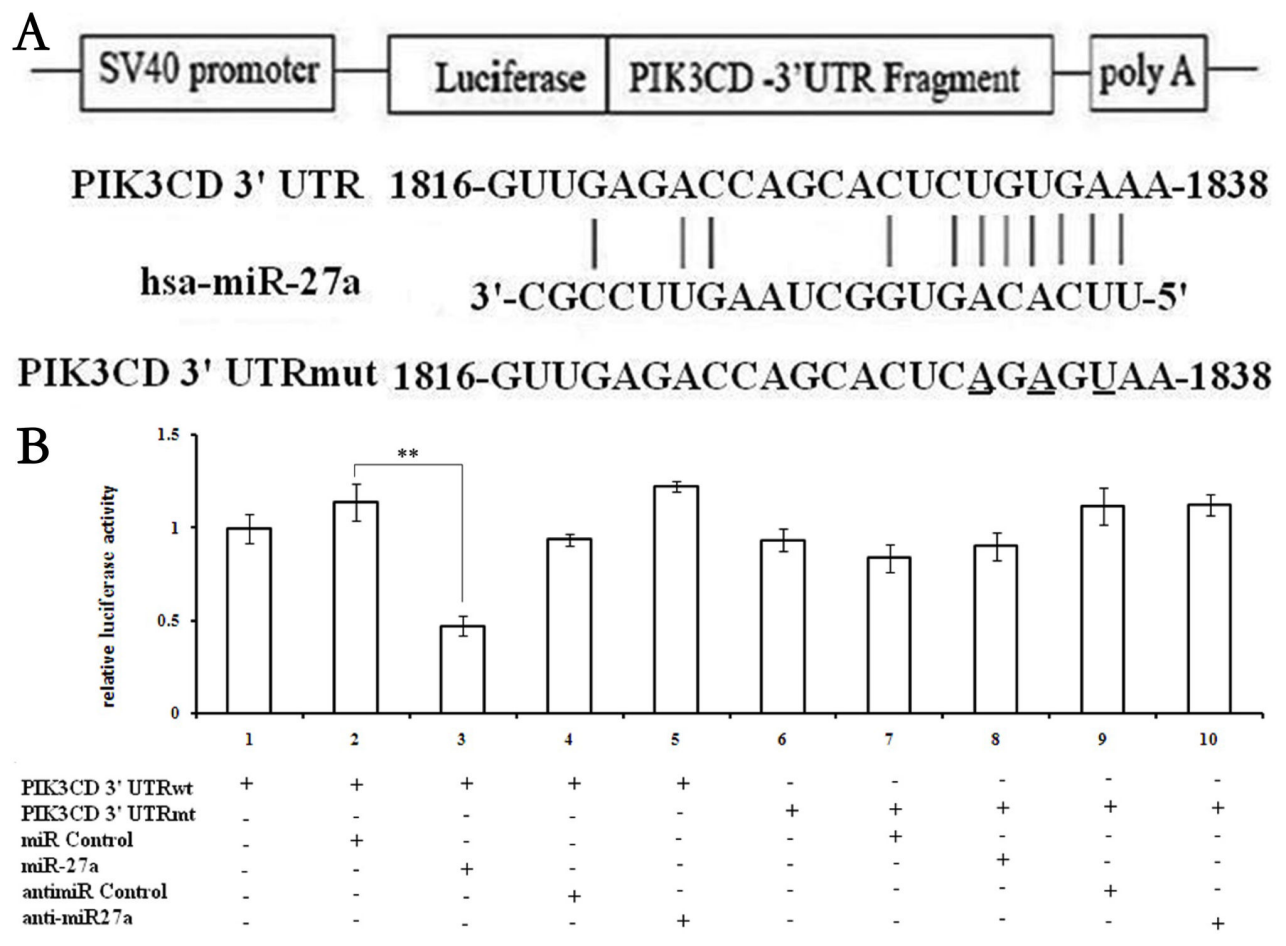
MiR-27a can inhibit PIK3CD by targeting the 3’-UTRs of PIK3CD. (A) Complementarity between miR-27a and the putative PIK3CD 3’-UTR target site. PIK3CD 3’-mut indicates the PIK3CD 3’-UTRs with three mutation sites (underlined) in miR-27a binding sites. (B) The relative luciferase activities of three independent experiments are shown. Error bars represent SEM; (**P<0.01).

We identified target sites of miR-27a using miRNA target predictionsoftware, known as miRTarG (http://mirtar.mbc.nctu.edu.tw), which indicated that the hsa-miR-27a target sites might be located in the 3’-UTR of PIK3CD mRNA. Moreover, RNA hybrid analysis provided further evidence of potential binding duplexes of the 3’-UTRs of PIK3CD with miR-27a (Figure 3A). To validate the computational prediction, relative luciferase activity was measured and was found to be markedly diminished (P<0.05) in cells cotransfected with miR-27a double-stranded mimics (Figure 3A). Moreover, mutation with three base pairs of the miR-27a binding sites of PIK3CD evidently abrogated the repression of luciferase activity due to miR-27a overexpression (Figure 3B).

### Overexpression of miR-27a induced apoptosis of NP cells

**Figure 4:**
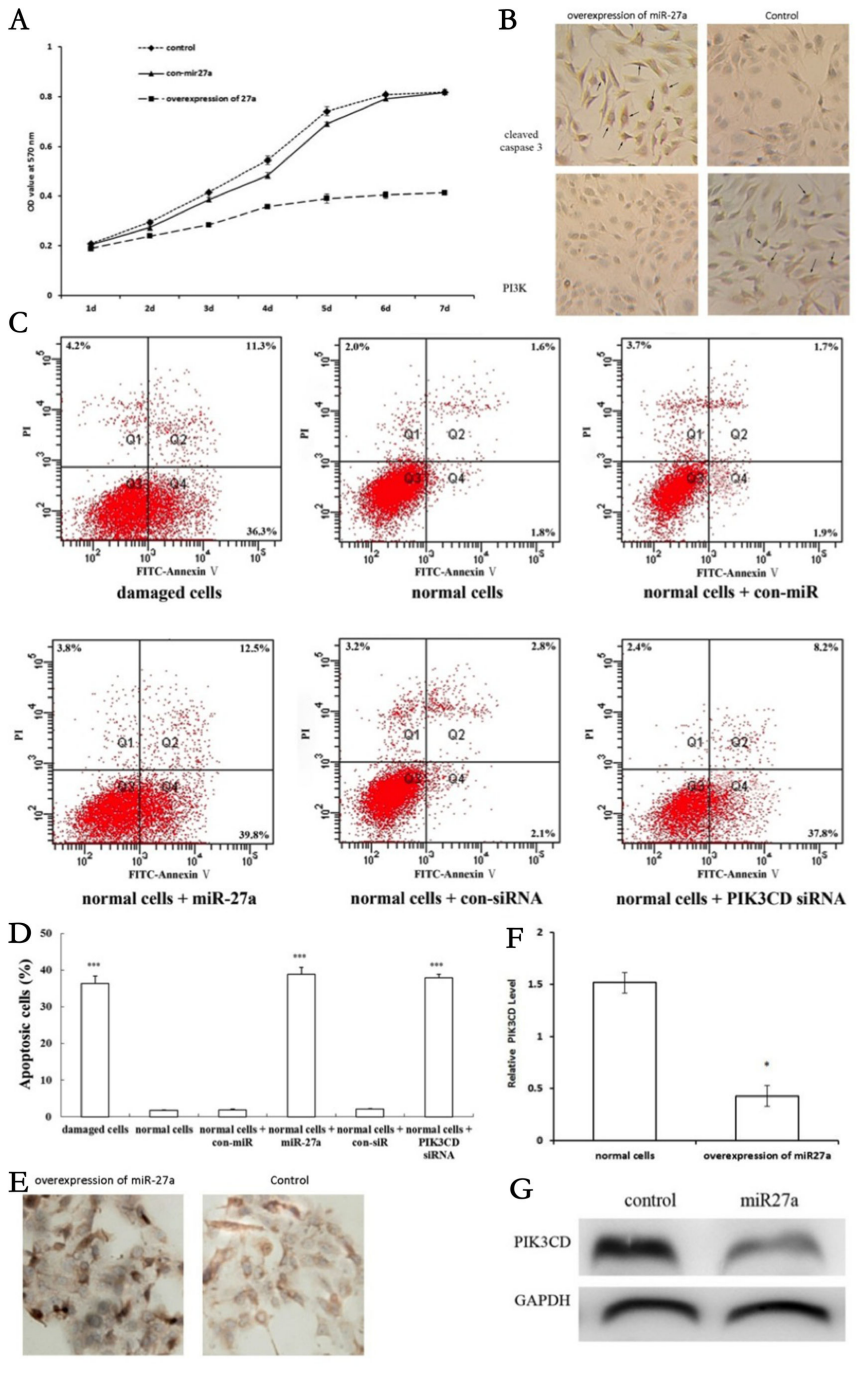
Overexpression of miR-27a in nucleus pulposus cells, and its function in the regulation of target proteins (A) Comparison of cell proliferation in various NP cell groups (*P<0.05). Data are representative of three experiments; error bars represent SEM. (B) Increased expression of cleaved caspase 3 and decreased expression of PIK3CD in the group overexpressing miR-27a compared to the control group (magnification ×200), respectively. Black arrows indicate positive-stained cells. (C) Contour diagram of FITC-Annexin V/PI FCM of human NP cells. The graphs represent typical results of cellular apoptosis; values represent the means of three experiments. Error bars represent SEM. (D) Results of cellular apoptosis was expressed as a fold change. Results are shown as mean ± SEM. Data are representative of three independent experiments (*P<0.05, ***P<0.001). (E) Increased numbers of nucleus pulposus cells were observed to undergo apoptosis in the group overexpressing miR-27a compared to the control group (magnification ×200). (F) (G) After 36 h, cellular protein lysates were prepared and PIK3CD expression was assessed by Western blot. GAPDH was used as an internal loading standard. Results are shown as mean ± SEM. Data are representative of three independent experiments (*P<0.05, **P<0.01).

We next investigated the influence of miR-27a on the phenotypes of NP cells. After 48h, cells transfected with miR-27a were found to grow more slowly than the negative control or the vehicle group (Figure 4 A). To further understand whether this reduced cell growth was due to apoptosis, we used the cleaved caspase 3 immunohistochemistry assay and the Annexin V/PI assay to assess apoptosis, respectively. High expression of cleaved caspase 3 was observed in cells transfected with miR-27a, while PI3K was decreased (Figure 4B). We observed an increase in cell apoptosis after miR-27a transfection,overexpression of both miR-27a and siRNA-PIK3CD increased apoptosis of NP cells (Figure 4C, D). Similarly, the percentage of cells with apoptotic nuclei obiviously increased in the miR-27a transfected group compared to controls when analyzed by PI staining (Figure 4C), and this increase in apoptosis compared with controls was confirmed by TUNEL staining (Figure 4E). Substantial decreases in the expression levels of PIK3CD after miR-27a transfection was observed (Figure 4F, G).

### Western blotting

**Figure 5:**
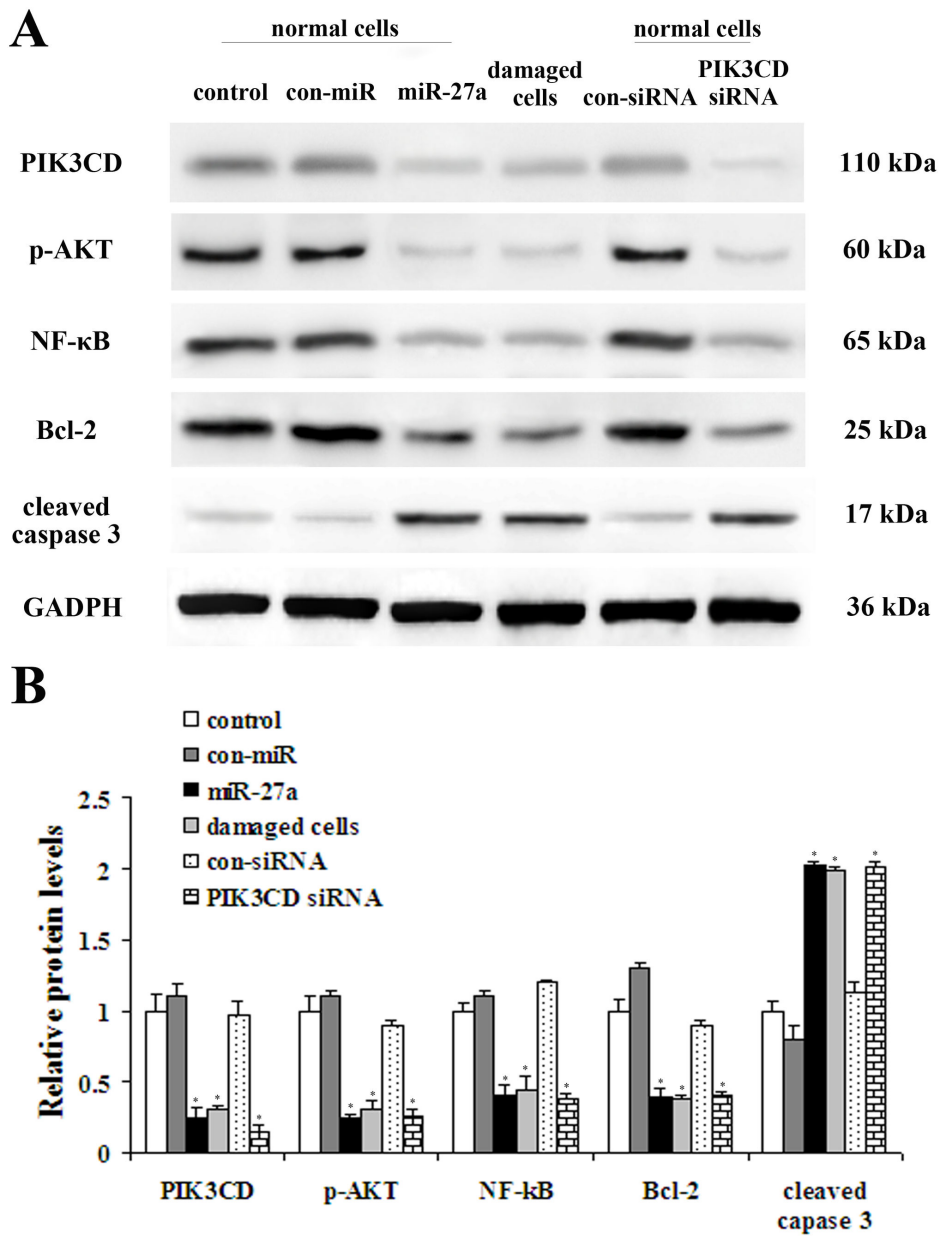
The role of PI3K/AKT pathway in miR-27a induced apoptosis. (A) Western blot analysis of total PI3KCD, p-AKT, NF-κB, Bcl-2 and cleaved caspase 3 protein levels inhuman NP cells with up-regulation and knockdown of miR-27a. (B) Quantification of band intensity in (A). Results are shown as mean ± SEM. Data are representative of three independent experiments (*P<0.05 vs control).

We investigated the role of PI3K/AKT pathway in miR-27a induced apoptosis by targeting PIK3CD and found that specific transient miR-27a expression reduced expression of PIK3CD, p-AKT, Bcl-2 and NF-κB in accordance with the results in damaged cells. Whereas knockdown of miR-27a with con-miR-27a led to overexpression of PIK3CD, p-AKT, Bcl-2 and NF-κB in NP cells (Figure 5). Caspase 3 is activated in the apoptotic cell both by intrinsic and extrinsic pathways. We examined the levels of cleaved caspase 3 and found that expression of miR-27a increased cleaved caspase 3 levels.

## Discussion

Nucleus pulposus cells play a very important role in maintaining the integrity of the IVD by producing type II collagen, aggrecan and other components of the extracellular-matrix (ECM). There is accumulating evidence that apoptosis of NP cells is likely to be involved in IDD.

It is an accepted fact that overload is the most significant factor in the induction of IVD degeneration [25-27]. Consistent with previously reported results [28], after a 36-hour induction by artificial compressed air in this study, proliferation of NP cells was markedly reduced. To further understand whether this reduction in cell growth was due to apoptosis, we measured apoptosis levels in cells following Annexin V/PI assay. We observed a time-dependent increase in cell apoptosis in the experimental group. Similarly, when assessed by PI staining, we found that the percentage of cells with apoptotic nuclei significantly increased in the experimental group compared to the control, suggesting that NP cells have been in apoptosis process when being treated with overload. It has been proven that overload accelerates the degeneration of IVD [25]. One notable *in vivo* study demonstrated that static and dynamic compression may induce different biologic responses in the IVD [27], suggesting that the apoptotic process could be regulated regardless of the underlying mechanisms.

It has been noted that both the extrinsic and intrinsic pathways play essential roles in the apoptosis of human NP cells [15]. The expression of FasL by NP cells could induce apoptosis of invading Fas-positive activated cytotoxic T-lymphocytes (CTLs), contributing to the immune privilege of NP. The FasL-Fas signalling pathway primarily pertains to several key proteins and caspases, the chief of which are Fas-associated death domaincontaining protein (FADD) and caspase 3 [29]. This death receptor pathway represents the extrinsic apoptotic signaling pathway, whereas the intrinsic pathway originates from the mitochondria and involves the activation of the Bcl-2 family [14]. Nucleus pulposus cells exhibit a robust expression of the phosphorylated survival protein AKT [11]. Activation of phosphoinositide-3 kinase (PI3K)/AKT signaling regulates cell growth, proliferation, migration and adhesion. PIK3CD acts as a suppressor of the activation of the PI3K/Akt signaling pathway [13], which is consistent with our result that lower expression of PIK3CD decreases the protein expression of phospho-Akt.

Accumulating evidence has shown that the apoptotic machinery is regulated by the newly defined small non-coding RNAs, miRNAs [17]. miRNAs control gene expression by targeting mRNAs and triggering either translation repression or RNA degradation [16]. Single miRNA is capable of regulating the expression of many target genes, whereas a target gene can also be regulated by several miRNAs [16,18]. The hundreds of miRNAs that have been identified regulate up to approximately 30% of all protein-encoding genes in human [16]. In animals, single-stranded miRNA binds the 3’-untranslated region (3’-UT R) of its target mRNA, termed miRNA response elements, causing the degradation or translational repression of the mRNA. These miRNAs act as key regulators in a wide variety of biological processes, including proliferation, differentiation, apoptosis, organ development and inflammatory diseases [22].

As a multi-functional miRNA, miR-27a is expressed in diverse tissues, and aberrant expression of miR-27a is found to be associated with various diseases [30-32]. In human NP cells, miR-base Target Database indicated that miR-27a target sites might be located in the 3’-UTR of PIK3CD mRNA. Moreover, RNA hybrid analysis provided further evidence demonstrating the potential binding duplexes of the 3’-UTRs of PIK3CD with miR-27a. To further confirm the role of miR-27a in apoptosis of NP cells, endogenous miR-27a was over-expressed in NP cells by transfection with Lenti-miR-27a, and these cells were then observed to grow more slowly than the negative control or vehicle group. We observed an increase in apoptosis after miR-27a transfection, suggesting that miR-27a positively regulates apoptosis of NP cells. To date, many target genes of miR-27a have been identified, such as PIK3CD, p-AKT, NF-κB, cleaved caspase3 and Bcl-2 [31]. We observed a specific transient miR-27a expression reduced expression of PIK3CD, p-AKT, Bcl-2 and NF-κB in accordance with the results in damaged cells, whereas knockdown of miR-27a with con-miR-27a led to overexpression of PIK3CD, p-AKT, Bcl-2 and NF-κB in NP cells. Caspase 3 is activated in the apoptotic cell both by intrinsic and extrinsic pathways. Our results show that overexpression of miR-27a inhibited PI3K expression, which was reversed by suppression of miR-27a, indicating that the PI3K/Akt pathway mediates the role of miR-27a in the apoptosis of NP cells. Taken together, these findings show that up-regulation of miR-27a expression might be responsible for the apoptosis of NP cells induced by PI3K, and that miR-27a interacts with its target gene PIK3CD to regulate the apoptotic process by facilitating activation of the PI3K/Akt signaling pathway.

In conclusion, we have identified PI3K as a novel target of miR-27a and show that upregulated miR-27a promotes apoptosis in human IDD by targeting PI3K, implicating a role of miR-27a in the etiology of IDD. Moreover, miR-27a could provide a potential therapeutic target for IDD using the lentiviral Had-miR-27a.
